# Removal of Off-Season Inflorescences Improves Fruit Retention, Kernel Quality, and Source–Sink Balance in Macadamia ‘O.C’

**DOI:** 10.3390/plants15203227

**Published:** 2026-10-21

**Authors:** Ya Ning, Yifan Li, Yuchun Chen, Tingmei Yang, Lichen Feng, Xiyong He, Hai Yue

**Affiliations:** Yunnan Provincial Woody Oil Technology Innovation Center, Yunnan Provincial Macadamia Agricultural Engineering Research Center, Yunnan Institute of Tropical Crops, Jinghong 666100, China; ningya@yitc.com.cn (Y.N.);

**Keywords:** carbohydrate status, fruit growth, fruit retention, kernel quality, *Macadamia integrifolia*, nutlet abscission, off-season flower removal

## Abstract

The effects of removing off-season inflorescences on retained fruit performance and whole-tree yield in macadamia remain insufficiently understood. To assess whether flower-removal timing influences reproductive and fruit development, we evaluated five treatments in seed-propagated macadamia trees using a randomized complete block design with five blocks and 25 trees. At the beginning of full bloom, all inflorescences of the designated October, January, February, or March flowering flush were manually removed from the whole tree; control trees received no flower removal. We measured the persistence of fruit-bearing tagged inflorescences, individual fruit growth, kernel traits, whole-tree yield, and leaf and branch carbohydrate status. Compared with the control, October flower removal increased the percentage of fruit-bearing tagged inflorescences before harvest from 12.9% to 18.3% (a 41.9% relative increase) and reduced their relative loss from 81.1% to 69.2% (an 11.9 percentage-point difference). These percentages describe inflorescence-level retention rather than flower-level fruit set. Individual fruit fresh weight at 180 days after flowering increased from 20.1 to 25.5 g, and kernel dry weight increased from 1.48 to 1.73 g. However, mean whole-tree fresh fruit yield with green husk was 35.98 kg tree^−1^ under October flower removal, compared with 54.75 kg tree^−1^ in the control (34.28% lower). By comparing removal of four flowering flushes, this study shows that favorable responses in retained inflorescences and sampled fruits do not necessarily translate into higher whole-tree yield. Flower removal also altered leaf and branch carbohydrate status, but these measurements do not demonstrate that carbohydrate competition caused the observed reproductive responses.

## 1. Introduction

Macadamia (*Macadamia* spp.) is a high-value nut crop owing to its high kernel oil content, desirable nutritional composition, and premium market value [1,2,3]. With the rapid expansion of the macadamia industry, improving fruit set stability, fruit development, and kernel quality has become a major objective of orchard management. However, macadamia is characterized by prolific flowering, low fruit set, and severe early fruit drop, resulting in substantial limitations to yield formation. Consequently, final productivity is influenced not only by pollination success but also by fruit retention, resource availability, and source–sink coordination during fruit development [2,4].

Macadamia produces racemose inflorescences bearing large numbers of flowers. Because of protandry and limited self-compatibility, successful fruit set largely depends on insect-mediated cross-pollination [5]. Previous studies have demonstrated that supplementary pollination and cross-cultivar pollen transfer can significantly affect fruit set, kernel development, and yield, highlighting the importance of pollination biology in reproductive success [6,7]. Nevertheless, substantial fruit abscission often occurs after initial fruit set, indicating that final fruit retention is determined not only by fertilization success but also by the capacity of developing fruits to acquire and maintain sufficient resources during subsequent growth [8,9].

In apple, sorbitol and sucrose synthesized in source leaves are transported to developing fruits, providing carbon for fruit growth and development [10]. Excessive flowering or the simultaneous presence of multiple cohorts of flowers and fruits may intensify competition among sink organs, thereby limiting carbohydrate availability and increasing nutlet abscission [11]. In macadamia, pruning-induced vegetative flushes have been shown to increase competition between vegetative growth and fruit development, resulting in reduced carbohydrate reserves, greater fruit abscission, and lower yield [12,13]. Girdling has been reported to reduce early fruit drop by improving carbohydrate availability and hormonal status, whereas carbohydrate depletion and associated changes in hormone metabolism and gene expression have been linked to fruit abscission under resource-limited conditions [14,15]. These studies collectively indicate that carbohydrate availability and source–sink coordination play crucial roles in fruit retention and development in macadamia.

In perennial fruit crops, crop-load regulation is widely used to optimize source–sink balance and improve fruit growth and quality. Fruit development depends on the supply of current photosynthates and remobilized reserves relative to the combined demand of reproductive and vegetative sinks [16,17,18]. Studies in apple and other fruit crops have shown that appropriate crop-load adjustment improves carbon allocation, fruit growth, and fruit quality, whereas excessive reproductive load intensifies assimilate competition and compromises fruit performance [19,20,21]. When multiple flowering flushes coexist, their different developmental stages may create overlapping periods of carbohydrate demand [22]. Removing a flowering flush may therefore reduce competition and increase assimilate availability for the remaining fruits, potentially supporting their retention and growth. However, this response depends on whether assimilate supply is limiting and whether the remaining sinks can use the resources made available. Higher sugar or starch concentrations in leaves and branches may also reflect reduced sink demand rather than increased carbon delivery to fruits [17]. Thus, carbohydrate concentrations provide information about source–sink status but do not directly measure assimilate transport. This framework supports examining flower-removal timing through the combined responses of tissue carbohydrate status, fruit retention, individual-fruit development, and whole-tree yield.

In major macadamia-growing regions of China, particularly Yunnan Province, some cultivars exhibit multiple flowering flushes within a season [23]. At the study site, off-season flowering of cultivar ‘O.C’ was observed in October, preceding the main flowering period. Flowers and young fruits derived from these off-season inflorescences may consume substantial photoassimilates and stored carbohydrates over an extended period and potentially compete with fruits developing during the main flowering season [11]. Moreover, different flowering cohorts experience distinct environmental conditions, including temperature, rainfall, and pollination environments, which may influence fruit development duration and fruit quality [23]. Consequently, the net effect of off-season flowering on the development of later fruits and whole-tree productivity remains unclear.

Previous studies in macadamia have primarily focused on pollination biology, pollen transfer, fruit abscission, pruning, and girdling [24,25,26,27]. Direct flower-removal experiments by Trueman and Turnbull [28] showed that reducing flower number increased the proportion of flowers retained as fruits, whereas severe reductions in flower number decreased final fruit numbers. These findings demonstrate that improved proportional retention does not necessarily correspond to increased fruit production. However, that study examined flower removal on racemes rather than comparing whole-tree removal of distinct seasonal flowering cohorts. The present study therefore compares removal of four flowering flushes and evaluates responses at both the retained-inflorescence and whole-tree levels, together with individual fruit traits and tissue carbohydrate status.

The present study extends previous work by comparing the removal of four distinct flowering flushes within one field experiment and jointly assessing responses at the tagged-inflorescence, individual-fruit, and whole-tree levels alongside leaf and branch carbohydrate status. October flowering precedes the main flowering season, and the resulting flowers and young fruits may draw on stored carbohydrates before the later flushes develop. Where their development overlaps, these reproductive cohorts may also compete for current assimilates. Evidence from macadamia pruning and girdling studies indicates that changes in competing sink demand and carbohydrate availability can influence fruit abscission [15,16,17]. On this basis, we hypothesized that removing the October flush would reduce early reproductive demand and, where carbohydrate supply limits development, improve resource availability for fruits retained from subsequent flushes, thereby favoring their retention, growth, and kernel quality. Therefore, the objectives of this study were to evaluate the effects of staged flower removal on fruit-bearing inflorescence persistence, nutlet abscission, fruit development, kernel quality, whole-tree fresh fruit yield, and leaf and branch carbohydrate status in macadamia cultivar ‘O.C’, and to identify the key source-related factors associated with fruit retention and quality formation. Whole-tree yield was assessed separately to determine whether improvements in the remaining fruits compensated for the removal of an entire flowering flush.

## 2. Results

### 2.1. Effects of Flower-Removal Timing on Inflorescence Retention and Nutlet Abscission

Reproductive characteristics differed among flowering flushes in macadamia cultivar ‘O.C’ (Table 1). The average number of retained nuts per inflorescence ranged from 1.23 to 4.10. October flowering exhibited the greatest number of retained nuts per inflorescence (4.10), followed by March (2.43) and February (2.12) flowering, whereas January flowering showed the lowest average number (1.23).

The distribution of fruit-bearing inflorescences also differed among flowering flushes (Table 1). October inflorescences had the highest proportion of inflorescences bearing ≥5 nuts (18.3%), whereas January inflorescences were predominantly characterized by single-nut retention (80.2%). February and March flowering showed intermediate distributions, with relatively high proportions of inflorescences bearing 2–4 nuts.

**Figure 1:**
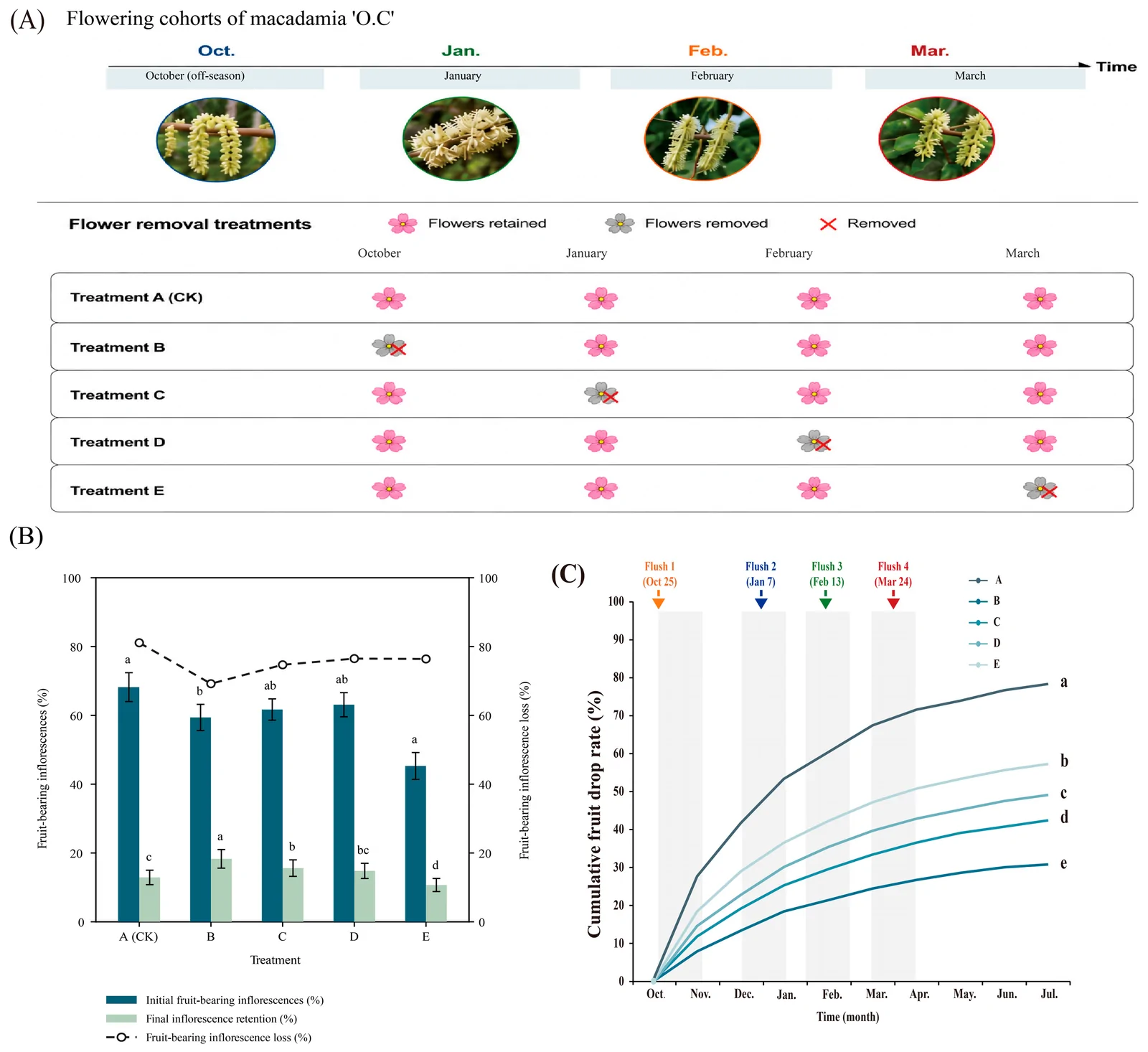
Flowering cohorts, flower-removal treatments, and reproductive responses in macadamia ‘O.C’: (**A**) Schematic of four flowering cohorts and five treatments (A, control; B–E, removal of October, January, February, and March inflorescences, respectively). (**B**) Initial and final fruit-bearing percentages of tagged inflorescences and relative loss (bars, mean ± SE, *n* = 5; dashed line, relative loss). (**C**) Cumulative nutlet abscission on tagged inflorescences (shaded bands, 15-day windows around operation dates). Different lowercase letters indicate significant differences among treatments (Tukey’s HSD, *p* < 0.05).

To further evaluate whether the timing of flower removal affected subsequent reproductive persistence, tagged inflorescences were monitored throughout fruit development. The initial and final retention percentages represent the proportions of tagged inflorescences bearing at least one fruit at the respective assessments. They are not flower-level fruit-set percentages. The relative loss of fruit-bearing inflorescences and count-based cumulative nutlet abscission were evaluated separately. The initial fruit-bearing inflorescence percentage ranged from 45.3% to 68.2% among treatments (Figure 1B). The control treatment (A) showed the highest initial percentage (68.2%), which was significantly higher than that of treatment B (59.4%). Treatments C and D showed intermediate values of 61.7% and 63.1%, respectively, and did not differ significantly from either A or B. Treatment E had the lowest initial percentage (45.3%).

In contrast, greater differences were observed in final inflorescence retention before harvest (Figure 1B). Treatment B, in which October off-season flowers were removed, showed the highest final retention rate (18.3%), significantly exceeding those of all other treatments. Treatments C and D showed intermediate retention rates of 15.6% and 14.8%, respectively, whereas the control treatment (A) showed a final retention rate of 12.9%. Treatment E exhibited the lowest final retention rate (10.7%). Relative to the control, October flower removal increased the final retention rate by 5.4 percentage points, equivalent to a relative increase of 41.9%. Treatment B had the lowest relative loss of fruit-bearing inflorescences between the early fruitlet stage and harvest (69.2%), representing an 11.9 percentage-point reduction relative to the control (81.1%). Treatments C and D showed fruit-bearing inflorescence loss percentages of 74.7% and 76.5%, respectively.

The count-based cumulative nutlet abscission curves showed different temporal patterns among treatments (Figure 1C). All treatments showed a rapid increase in cumulative nutlet abscission during the early stages of fruit development, followed by a gradual stabilization at later stages. Treatment B showed lower cumulative nutlet abscission than the control over the observation period. These curves describe changes in the number of attached nutlets and are distinct from the loss of fruit-bearing inflorescences reported in Figure 1B.

### 2.2. Effects of Staged Flower Removal on Fruit Growth Dynamics

**Figure 2:**
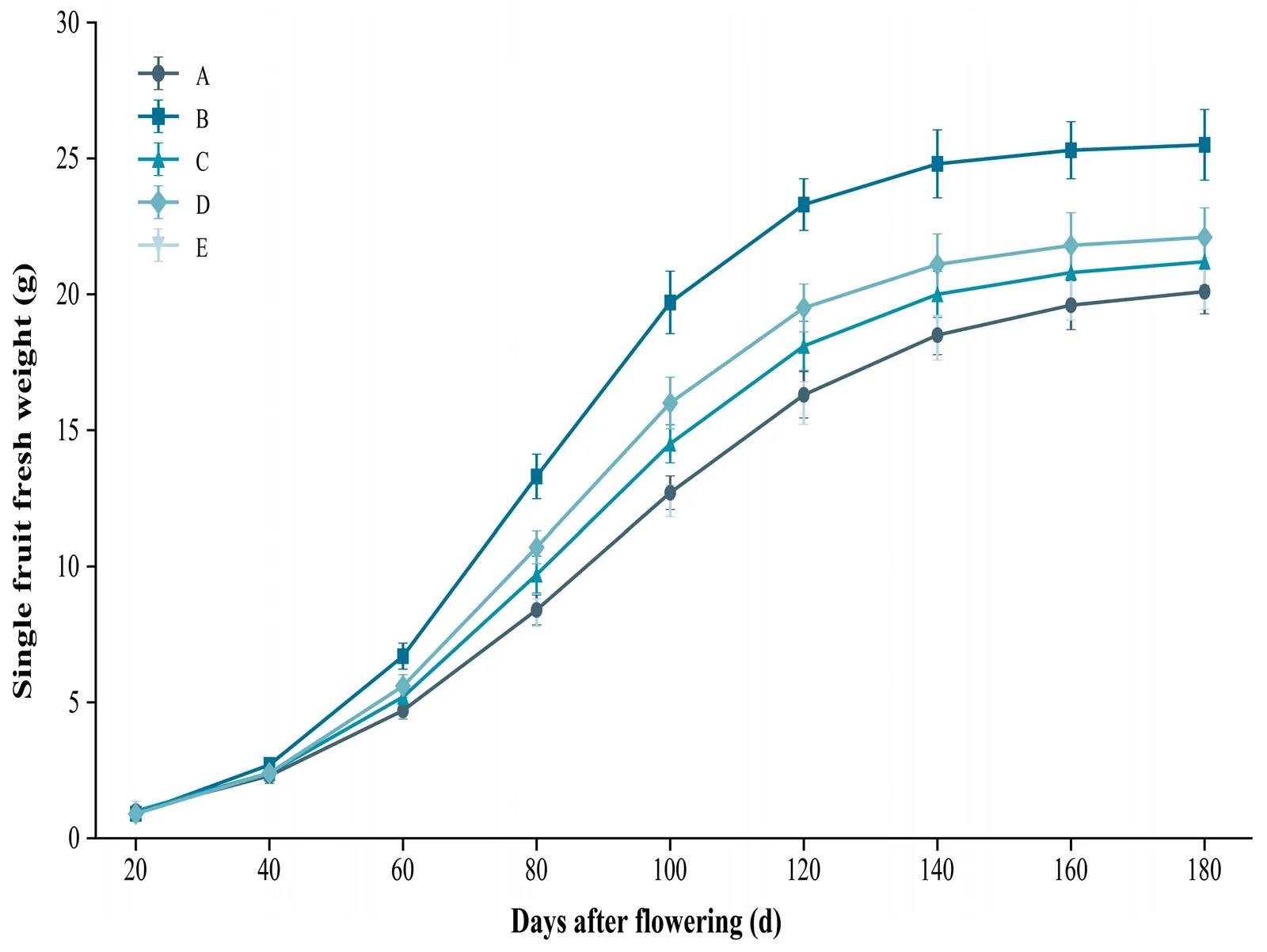
Effects of staged flower removal on fruit fresh weight accumulation dynamics in macadamia cultivar ‘O.C’. Values are means ± SE (*n* = 5).

Staged flower-removal treatments significantly affected the fresh weight growth dynamics of individual fruits in macadamia cultivar ‘O.C’ (Figure 2). Fruit fresh weight increased continuously throughout development and exhibited a typical sigmoid growth pattern. During the early stage of fruit development (20–60 DAF), fruit growth was relatively slow, followed by a rapid enlargement phase between 60 and 120 DAF and a gradual decline in growth rate toward fruit maturity.

Clear differences in fruit growth were observed among treatments. At maturity (180 DAF), treatment B produced the highest fruit fresh weight (25.5 g), followed by treatments D (22.1 g) and C (21.2 g), whereas treatments E and A showed lower values of 20.4 and 20.1 g, respectively. Fruit fresh weight in treatment B was 26.9% higher than that in the control at 180 DAF. Throughout fruit development, treatment B maintained a greater fruit fresh weight than the other treatments, particularly during the rapid enlargement phase.

The logistic model provided a good fit for fruit growth dynamics under all treatments, with coefficients of determination (R^2^) ranging from 0.981 to 0.994 (Table 2). The estimated parameters differed among treatments, with treatment B showing a higher theoretical maximum fruit weight (*W_max_*), faster growth rate (*k*), and earlier inflection point (*t*_0_) than the control.

Treatment B exhibited the highest *W_max_* (25.6 ± 0.7 g) and k value (0.056 ± 0.003 d^−1^), together with the earliest estimated inflection point (78.5 ± 1.8 DAF). Treatments C and D showed intermediate parameter estimates, whereas treatments A and E exhibited lower *W_max_* values and later estimated inflection points. The corresponding control estimates were 20.5 g for *W_max_*, 0.043 d^−1^ for k, and 88.5 DAF for *t*_0_ (Table 2).

### 2.3. Effects of Staged Flower Removal on Fruit Quality Traits and Whole-Tree Yield

**Figure 3:**
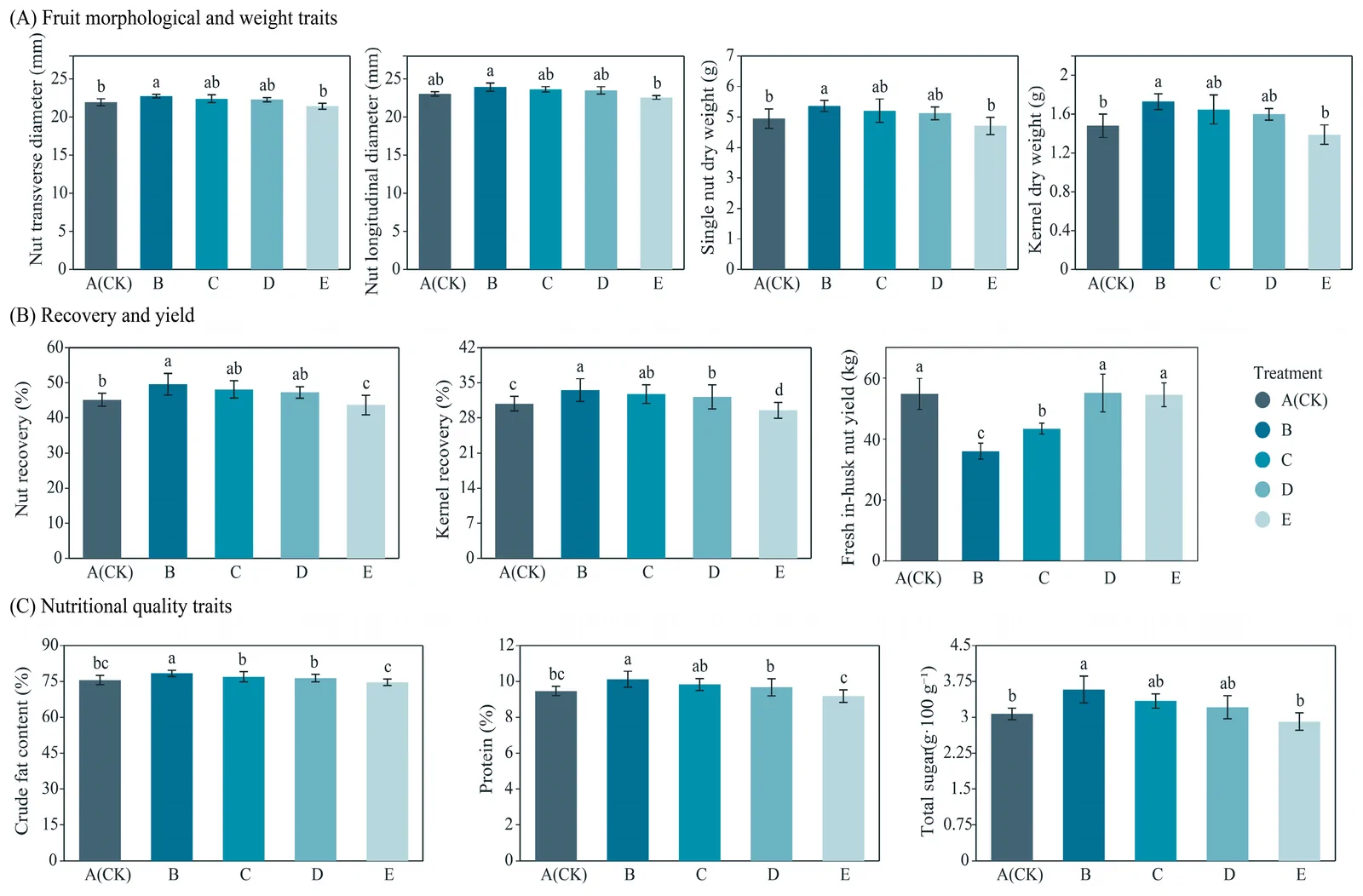
Effects of inflorescence removal timing on nut traits, yield, and kernel nutritional quality in macadamia ‘O.C’. (**A**) Fruit morphological and weight traits. (**B**) Recovery and yield. (**C**) Kernel crude fat, protein, and total sugar contents. Treatments were A (CK), no inflorescence removal; B, October inflorescence removal; C, January inflorescence removal; D, February inflorescence removal; and E, March inflorescence removal. Bars represent means ± SE (*n* = 5 trees). Different lowercase letters indicate significant differences among treatments within each trait (Tukey’s HSD test, *p* < 0.05).

Staged flower-removal treatments significantly affected fruit morphology, yield-related traits, and nutritional quality in macadamia cultivar ‘O.C’ (Figure 3).

For fruit morphological traits (Figure 3A), treatment B exhibited the highest values for nut transverse diameter (22.76 mm), nut longitudinal diameter (23.92 mm), dry nut weight (5.36 g), and kernel dry weight (1.73 g), all of which were significantly greater than those of the control treatment (A) and treatment E. Kernel dry weight in treatment B was 16.9% higher than the control value of 1.48 g. Treatments C and D showed intermediate values and did not differ significantly from treatment B, whereas treatment E exhibited the lowest values for all morphological traits.

Recovery-related traits showed similar treatment-dependent patterns (Figure 3B). Treatment B produced the highest kernel recovery (33.56%), followed by treatments C (32.74%) and D (32.18%), which were not significantly different from treatment B. The control treatment exhibited a kernel recovery of 30.84%, whereas treatment E showed the lowest value. Similar trends were observed for nut recovery, with treatment B reaching 49.58%, compared with 48.12%, 47.26%, and 45.16% for treatments C, D, and A, respectively.

Nutritional quality was also significantly affected by flower-removal treatments (Figure 3C). Treatment B exhibited the highest crude fat (78.34%), protein (10.12%), and total sugar contents (3.58 g·100 g^−1^), significantly exceeding those of the control and treatment E. Treatments C and D showed intermediate values and did not differ significantly from treatment B. The lowest crude fat (74.62%), protein (9.18%), and total sugar contents were recorded in treatment E.

Mean whole-tree fresh fruit yield, measured with the green husk intact, was 54.75 ± 5.10, 35.98 ± 2.60, 43.37 ± 1.80, 55.09 ± 6.20, and 54.46 ± 3.90 kg tree^−1^ for treatments A, B, C, D, and E, respectively (mean ± SE, *n* = 5 trees per treatment). Relative to the control, mean yield was 34.3% lower in treatment B and 20.8% lower in treatment C, whereas treatments D and E differed from the control by +0.6% and −0.5%, respectively. These percentage changes describe differences in treatment means.

### 2.4. Responses of Source-Related Traits and Source–Sink Balance During Fruit Enlargement

**Figure 4:**
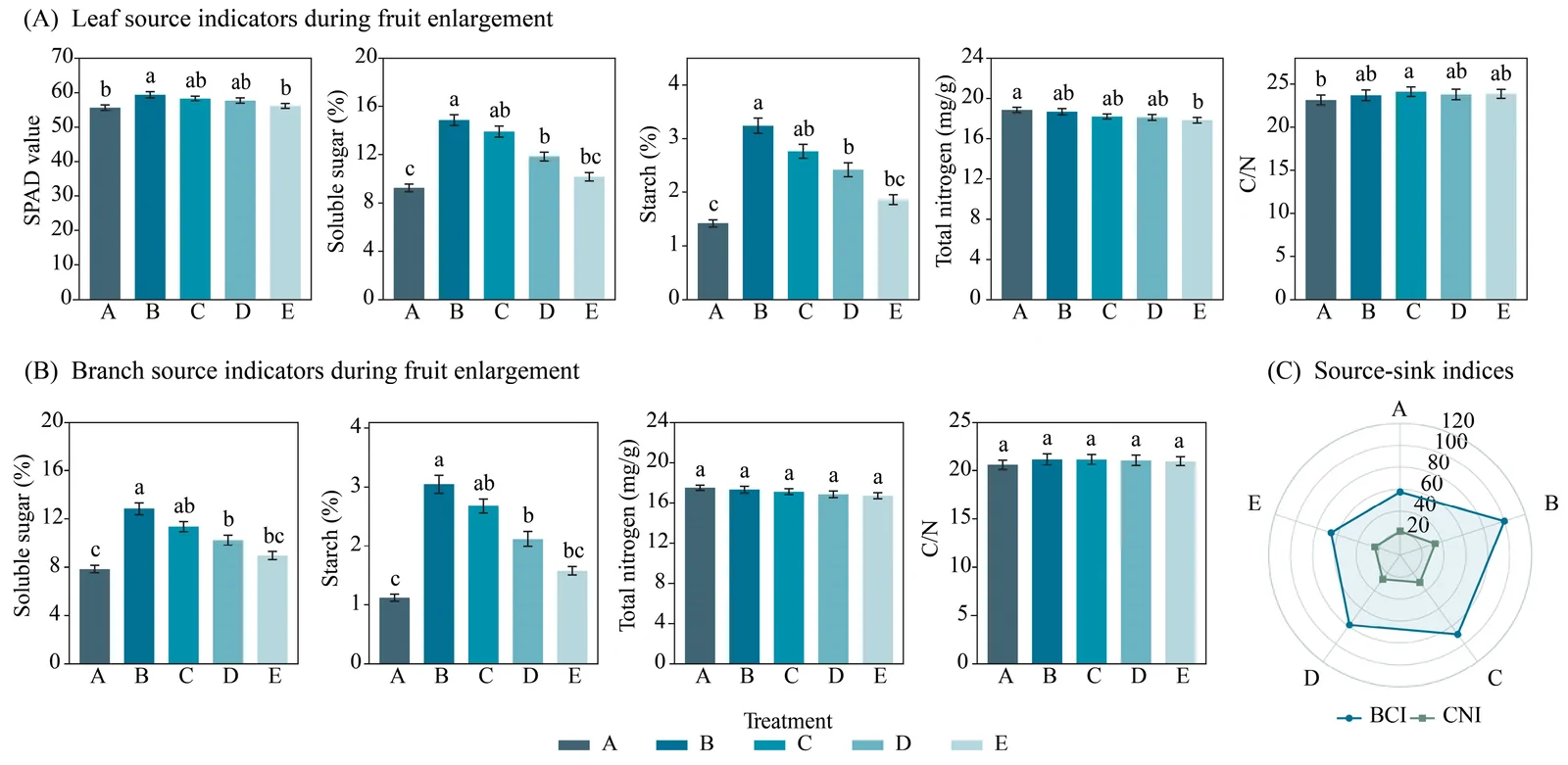
Effects of staged flower removal on source-related traits and source–sink indices during fruit enlargement in macadamia ‘O.C’: (**A**) Leaf source indicators during fruit enlargement. (**B**) Branch source indicators during fruit enlargement. (**C**) Source–sink indices. Values are means ± SE (*n* = 5). Different lowercase letters indicate significant differences among treatments (Tukey’s HSD, *p* < 0.05). BCI, branch–leaf carbohydrate index; CNI, carbon–nitrogen index.

Staged flower-removal treatments significantly affected several leaf and branch physiological traits, including soluble sugar and starch concentrations, in macadamia cultivar ‘O.C’ during the fruit enlargement stage (Figure 4).

For leaf source-related traits (Figure 4A), SPAD values varied only slightly among treatments, although treatment B exhibited the highest value (59.4), which was significantly higher than those of treatments A, D, and E, but did not differ significantly from treatment C. In contrast, leaf carbohydrate concentrations exhibited much greater variation among treatments. Treatment B showed the highest soluble sugar (15.02%) and starch (3.18%) contents, while treatment C exhibited comparable values (13.74% and 2.82%, respectively) and did not differ significantly from treatment B. Compared with the control values of 9.24% for soluble sugars and 1.42% for starch, treatment B showed relative increases of 62.6% and 123.9%, respectively. Treatments D, A, and E exhibited progressively lower carbohydrate concentrations. Leaf total nitrogen content, however, varied only slightly among treatments (17.82–18.84 mg g^−1^), with no significant differences detected. The leaf C/N ratio followed a pattern similar to carbohydrate accumulation, peaking in treatment B (35.42), followed by treatment C (31.96).

Branch source-related traits exhibited trends comparable to those observed in leaves (Figure 4B). Treatment B recorded the highest branch soluble sugar (12.84%) and starch (3.05%) contents, followed by treatment C (11.52% and 2.62%, respectively), with no significant difference between the two treatments. Lower values were observed in treatments D and E, whereas treatment A consistently showed the lowest carbohydrate concentrations. Branch total nitrogen content varied within a narrow range (16.72–17.50 mg g^−1^) and did not differ significantly among treatments. The branch C/N ratio followed the same trend as carbohydrate accumulation, with treatment B exhibiting the highest value (31.84), followed by treatment C (28.46).

The composite source–sink indices (BCI and CNI) showed similar variation patterns to the measured carbohydrate-related traits, with treatment B exhibiting the highest values (Figure 4C). These indices were used as exploratory integrative parameters to summarize treatment-associated variation in carbohydrate accumulation and carbon–nitrogen status.

### 2.5. Relationships Between Source–Sink Balance Indices and Fruit Traits and Identification of Key Contributing Factors

**Figure 5:**
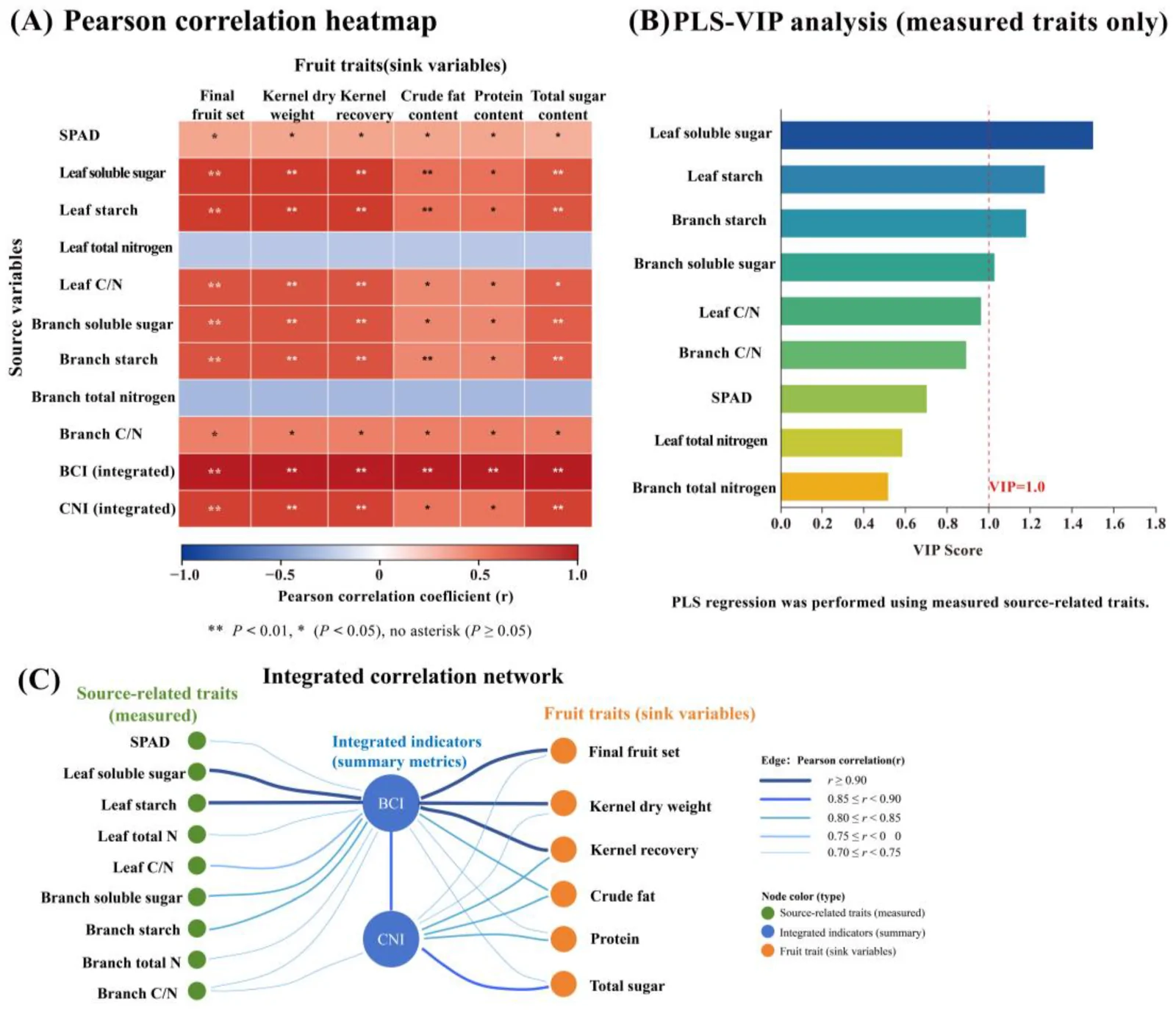
Relationships between source-related traits and fruit traits in macadamia cultivar ‘O.C’: (**A**) Pearson correlation heatmap based on tree-level observations (*n* = 25). (**B**) Variable importance in projection (VIP) ranking derived from partial least squares (PLS) regression using measured source-related traits. (**C**) Integrated correlation network illustrating relationships among source-related traits and fruit traits based on Pearson correlation coefficients and PLS analysis. * and ** indicate significance at *p* < 0.05 and *p* < 0.01, respectively.

Pearson correlation analysis revealed significant associations between source-related traits and fruit retention, kernel development, and nutritional quality in macadamia cultivar ‘O.C’ (Figure 5A). Correlation analyses were performed using tree-level mean values from 25 individual trees as independent observations (*n* = 25).

Among the evaluated source-related traits, carbohydrate-related parameters exhibited strong positive relationships with fruit performance. The branch–leaf carbohydrate index (BCI), used here as a descriptive summary of leaf and branch carbohydrate concentrations, showed strong positive associations with final inflorescence retention (r = 0.91, *p* < 0.01), kernel dry weight (r = 0.89, *p* < 0.01), kernel recovery (r = 0.87, *p* < 0.01), crude fat content (r = 0.84, *p* < 0.01), protein content (r = 0.80, *p* < 0.01), and total sugar content (r = 0.86, *p* < 0.01). Similar positive correlations were observed for leaf soluble sugar, leaf starch, branch soluble sugar, branch starch, and leaf and branch C/N ratios (r = 0.64–0.89). In contrast, leaf and branch total nitrogen contents showed relatively weak correlations with fruit traits (r = 0.28–0.41), whereas SPAD values exhibited moderate positive relationships (r = 0.58–0.68). These coefficients describe associations within the experimental dataset.

To explore the relative contributions of source-related traits to variation in fruit characteristics, partial least squares (PLS) regression was conducted (Figure 5B). Leaf soluble sugar, leaf starch, branch starch, branch soluble sugar, and C/N ratios showed relatively higher VIP values. Variables with VIP values greater than 1.0 were considered relatively important variables within the fitted model. These rankings describe variable contributions in the present dataset and do not establish predictive performance for independent observations.

The integrated correlation network combined Pearson correlation coefficients with PLS-VIP results to visualize the relationships among source-related traits and fruit characteristics (Figure 5C). Carbohydrate-related traits occupied central positions in the network, whereas nitrogen-related traits exhibited relatively weaker associations. BCI and CNI showed similar variation patterns with measured source traits and were used as integrated indicators to summarize treatment-associated changes in source status.

### 2.6. Multivariate Analysis of Treatment-Associated Variation Following Staged Flower Removal

Principal component analysis (PCA) was performed to summarize variation in inflorescence retention, fruit development, kernel quality, and leaf and branch physiological traits among staged flower-removal treatments. The first three principal components explained 99.96% of the total variance, with PC1, PC2, and PC3 accounting for 94.70%, 4.84%, and 0.42%, respectively. PC1 accounted for most of the variance in the variables included in the analysis.

**Figure 6:**
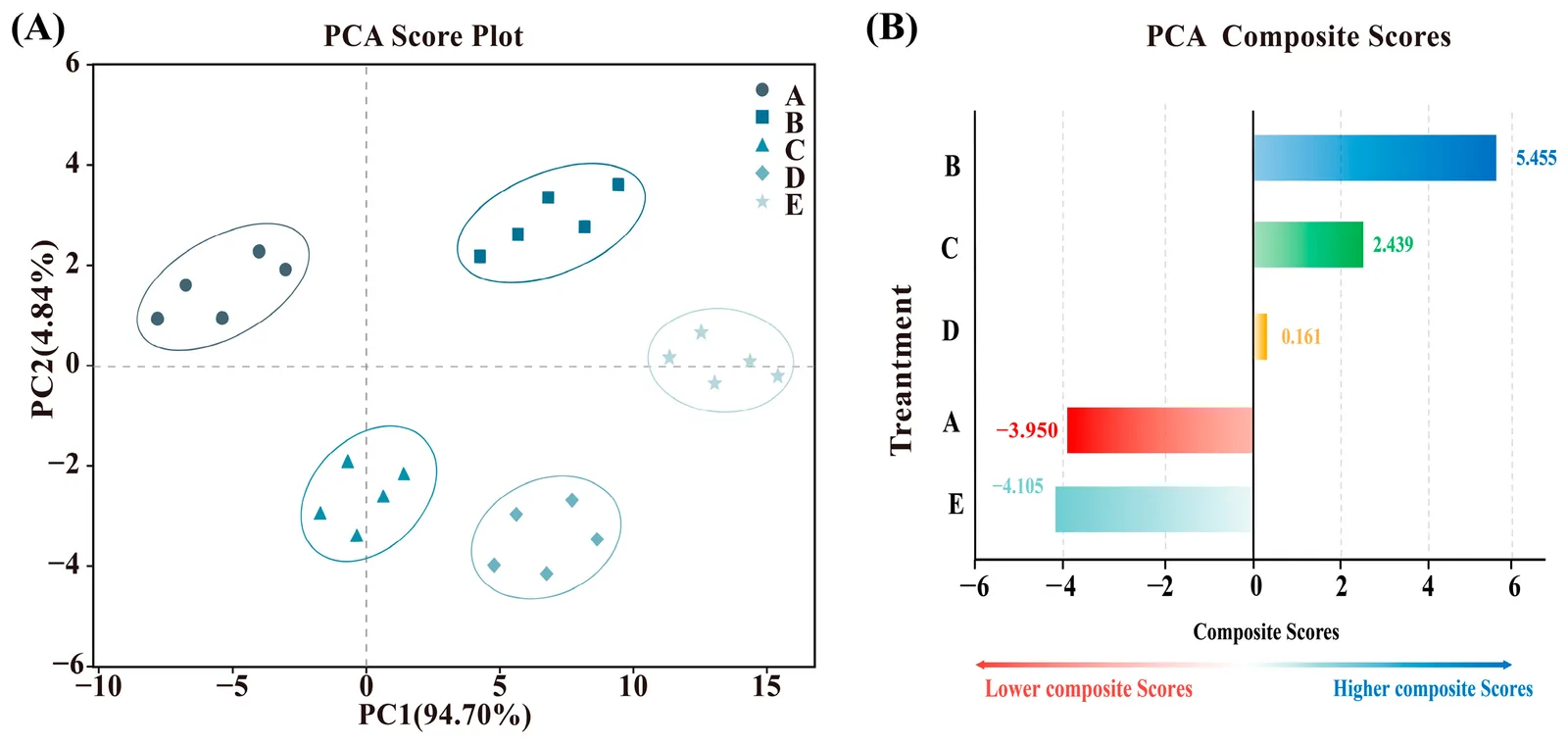
Principal component analysis (PCA) of the overall responses to staged flower-removal treatments in macadamia cultivar ‘O.C’: (**A**) PCA score plot showing the distribution of treatments in the PC1–PC2 space. (**B**) Comprehensive PCA scores calculated from the variance contribution rates of the principal components.

The PCA score plot showed differences in treatment positions in the PC1–PC2 space (Figure 6A). Treatment B was located in the positive region of PC1 and was separated from the other treatments, indicating a distinct overall response pattern associated with October off-season flower removal. Treatment C was also distributed on the positive side of PC1, whereas treatments A (CK), D, and E showed relatively lower PC1 scores. These positions summarize differences across the variables included in the PCA.

Comprehensive PCA scores based on the retained principal components showed differences in the overall response patterns among treatments (Figure 6B). Treatment B exhibited the highest comprehensive score, followed by treatments C and D, whereas treatments A and E showed relatively lower scores. These results indicate that removal timing influenced the integrated response of fruit-related and source-related traits, with October flower removal producing a distinct multivariate response pattern under the experimental conditions.

## 3. Discussion

### 3.1. Staged Flower Removal Improves Fruit Retention by Alleviating Resource Competition

Macadamia is characterized by abundant flowering, low fruit set, and severe early fruit drop. Consequently, final yield formation depends not only on successful pollination and fertilization but also on the ability of developing fruitlets to continuously acquire sufficient assimilates during early development [2,27]. In the present study, the control treatment exhibited a relatively high initial inflorescence retention rate and greater reproductive loss. In contrast, removal of October off-season flowers resulted in the highest final inflorescence retention rate and the lowest cumulative retention loss, although it did not produce the highest initial inflorescence retention rate. These results indicate that staged flower removal primarily influenced subsequent reproductive persistence rather than early reproductive establishment.

A possible explanation is that off-season flowers may act as competing sink organs before and during early fruit development [29,30]. Flowering and early reproductive growth require substantial assimilates, and excessive reproductive sinks may increase competition for stored carbohydrates and current photosynthates before stable fruit development is established [31,32]. Removing October inflorescences eliminates both their immediate resource demand and the potential demand of fruits that would subsequently develop from them. This reduction in reproductive load could alter the resources available to retained flowering cohorts and their developing fruits. However, the treatment also changes the composition of retained flowering cohorts, so differences in their developmental timing and retention characteristics may contribute to the observed response. Previous studies have shown that flower removal, girdling, and dry matter accumulation are closely associated with fruit retention in macadamia [28], while vegetative flushes induced by pruning can increase carbohydrate competition and fruit abscission [13]. The present results are therefore consistent with reduced competition among reproductive sinks, but do not directly establish the amount or direction of assimilate redistribution.

Trueman and Turnbull [28] found that flower removal increased the proportion of flowers retained as fruits, while final fruit numbers decreased only after severe reductions in flower number. Our observation of greater final retention among tagged racemes following October flower removal is broadly consistent with a compensatory retention response. However, their flower-level measurements differ from the raceme-level retention measure used here, and the response magnitudes are therefore not directly comparable. Moreover, our treatment removed an entire flowering cohort across the tree, and mean whole-tree fresh fruit yield was lower despite improved retention among the remaining racemes. This difference highlights the importance of distinguishing retention efficiency from total production. McFadyen et al. [13] also reported increased fruit abscission and reduced stem carbohydrates following post-pruning shoot growth. Although both studies implicate competition among sinks, pruning removes source leaves and stimulates vegetative growth, whereas our treatment directly removes reproductive sinks. Consequently, contrasting responses may reflect differences in both source removal and the type of competing sink.

Fruit drop dynamics further indicated that the major fruit abscission period occurred approximately 30–50 d after flowering. This period likely represents a critical developmental window during which fruitlets are highly sensitive to carbohydrate availability. Similar observations have been reported in citrus, where fruit abscission is strongly associated with carbohydrate shortage, ethylene signaling, and hormonal regulation [33,34,35]. In macadamia, Yang and Xiang [14] induced carbohydrate starvation through girdling combined with defoliation and observed decreases in fruit carbohydrate concentrations before extensive fruit shedding, together with changes in endogenous hormones and related gene expression. Their experiment provides evidence linking disrupted carbon supply to fruit abscission, whereas the present study examined the consequences of removing a reproductive cohort. Our findings are compatible with carbohydrate-related regulation of retention, but do not demonstrate that flower removal reversed the hormonal or molecular responses observed under starvation stress. In addition, our carbohydrate measurements at 120 DAF were made substantially later than the principal abscission period and therefore cannot directly explain the early retention response.

### 3.2. Staged Flower Removal Improves Fruit Enlargement and Kernel Quality Formation Under Improved Source–Sink Conditions

The effects of staged flower removal extended beyond reproductive persistence and were reflected in fruit growth dynamics and kernel quality formation. Removal of October off-season flowers significantly increased fruit growth rate, theoretical maximum fruit weight, kernel dry weight, kernel recovery, crude fat content, protein content, and total sugar content. Logistic model analysis further showed that treatment B exhibited the highest *W_max_* and growth rate constant (k), together with the earliest inflection point (*t*_0_), indicating enhanced fruit enlargement capacity during development.

Fruit growth is regulated by the interaction between source supply and sink demand. According to source–sink theory, fruit development depends on current photosynthetic assimilates, remobilized carbohydrate reserves, and fruit sink strength [36]. Studies in apple and other fruit crops have demonstrated that optimized crop load management can improve carbon allocation, fruit growth, and quality formation, whereas excessive reproductive load intensifies assimilate competition and reduces fruit quality [10,19,37]. In the present study, the greater fresh weight and kernel dry weight of sampled fruits following October flower removal are consistent with greater resource availability per retained fruit. Nevertheless, these responses do not establish an increase in whole-tree assimilate production. Higher carbohydrate concentrations in sampled tissues could reflect reduced utilization following sink removal, greater supply, or both. Consequently, improved individual fruit growth should be interpreted alongside the lower mean whole-tree yield, rather than as evidence of an overall increase in tree productivity.

Kernel filling and lipid accumulation may represent additional mechanisms underlying quality improvement. Macadamia kernel quality largely depends on dry matter accumulation and oil biosynthesis, both of which require substantial carbon input [1,3,38]. Transcriptomic and lipidomic studies have shown that fatty acid biosynthesis and oil body formation are central processes during kernel development [39]. The higher kernel dry weight under October flower removal indicates greater dry matter accumulation per sampled kernel. The accompanying difference in crude fat concentration suggests that kernel composition also changed. These observations are compatible with altered carbon availability during kernel development, but they do not demonstrate an increased rate of lipid biosynthesis. Because assimilate transport, lipid synthesis rates, and the activities of associated enzymes were not measured, enhanced carbon delivery to kernels remains a physiological explanation to be tested.

The biological relevance of the treatment differences depends on the scale of assessment. October flower removal increased final inflorescence retention from 12.9% to 18.3%, corresponding to an increase of 5.4 percentage points, or 41.9% relative to the control. This indicates greater persistence of fruit-bearing racemes among the retained flowering flushes, rather than a demonstrated increase in flower-level fruit set. Fruit fresh weight at 180 DAF and kernel dry weight were also higher by 26.9% and 16.9%, respectively, indicating greater growth and kernel dry matter accumulation per sampled fruit. However, mean whole-tree fresh fruit yield with the green husk intact was 34.3% lower under October flower removal. Thus, improved retention among retained racemes and larger individual fruits did not translate into higher mean whole-tree yield in this season. These contrasting responses are consistent with a possible trade-off between retaining reproductive sites and supporting the development of individual fruits, although the absence of flush-specific harvest totals prevents the contribution of each flowering cohort to whole-tree yield from being resolved.

### 3.3. Carbohydrate Status Is Associated with Inflorescence Retention and Kernel Development

Flower-removal treatments altered leaf and branch carbohydrate status alongside changes in reproductive and fruit traits. Although flower-removal treatments had little effect on total nitrogen concentration in leaves and branches, they significantly altered soluble sugar, starch, C/N ratio, BCI, and CNI. These responses indicate that carbohydrate-related traits varied more strongly among treatments than tissue nitrogen concentration. According to source–sink theory, fruit development depends on the balance among assimilate production, stored carbohydrate availability, and sink demand [18,40]. The increases in leaf soluble sugar and starch concentrations under October flower removal were substantial relative to the control, at 62.6% and 123.9%, respectively. These differences indicate altered carbohydrate status in the sampled leaves during fruit enlargement. However, higher tissue carbohydrate concentrations can reflect changes in both carbohydrate supply and utilization, and therefore cannot alone demonstrate increased photosynthesis or enhanced assimilate delivery to fruits. Their concurrence with greater individual fruit growth supports an association between carbohydrate status and fruit development, while the underlying allocation processes remain to be verified.

In the present study, removal of October off-season flowers resulted in higher leaf soluble sugar and starch concentrations than the control. Branch carbohydrate concentrations also differed among treatments, although their responses should be considered separately from those of leaves. The concurrent increase in leaf carbohydrate concentrations and final inflorescence retention is consistent with an association between carbohydrate status and retained fruit development [41]. Similar relationships between carbohydrate availability, reproductive sink strength, and fruit retention have been reported in perennial fruit crops [28,42]. However, because carbohydrate fluxes and organ-specific assimilate transport were not directly measured in the present study, the observed responses should be interpreted as associations with tissue carbohydrate status rather than direct evidence of assimilate redistribution. Moreover, the measurements at 120 days after flowering cannot directly explain the earlier nutlet-abscission responses.

The relatively stable total nitrogen concentrations across treatments further indicate that nitrogen concentration alone was unlikely to explain the observed differences in fruit performance. The carbohydrate-related responses provide physiological context for the treatment differences, but do not establish that greater carbohydrate availability caused improved retention or kernel development [43,44].

BCI integrates leaf and branch soluble sugar and starch concentrations, and correlates with inflorescence retention and kernel traits to describe carbohydrate-related variation in this experiment. These associations do not confirm predictive performance or causality: BCI is mathematically derived from the measured carbohydrate variables, making its correlations with these components partially inherent and non-independent for validation. Its links to fruit traits may also reflect shared responses to flower-removal treatments. Critically, BCI only quantifies tissue-level concentrations, not whole-tree carbohydrate reserves or assimilate transport. Validation across independent growing seasons, sites and cultivars is required before BCI can be adopted as a reliable field predictor or management tool; its dataset-specific normalization also prevents interpretation as a universal physiological threshold. CNI is similarly a descriptive index with unproven predictive utility.

PLS analysis quantified the relative contributions of source-related variables to fruit trait variation. VIP rankings alone do not establish model robustness or predictive accuracy. Without documented model performance and independent validation, these rankings are only valid within the current dataset and cannot support extrapolation.

PCA captured treatment differences across selected reproductive, fruit-quality and physiological variables. High PC1 variance explained does not inherently identify its underlying biological processes, which requires explicit examination of variable loadings. Composite PCA treatment scores are contingent on variable selection and scaling, and cannot be interpreted as a universal commercial performance ranking, as whole-tree yield was independently evaluated.

From a commercial perspective, improved inflorescence retention and individual fruit traits did not increase mean whole-tree yield. October flower removal reduced fresh nut-in-husk yield to 35.98 kg tree^−1^, a 34.28% decrease relative to the 54.75 kg tree^−1^ control. Whether kernel trait improvements can economically offset this yield loss remains untested, as whole-tree marketable kernel yield, labor inputs and net economic returns were not measured. These single-season results do not support recommending complete October inflorescence removal for commercial production.

This single-orchard, single-season study on seed-propagated trees limits generalization to other plant materials and production environments. Treatment responses may also reflect differences among retained flowering cohorts, whose individual yield contributions cannot be resolved without cohort-specific harvest data. Future trials should compare full and partial flower removal across multiple growing seasons, sites and genotypes, measuring cohort-specific yield, whole-tree marketable kernel yield, labor costs, and subsequent flowering and production. Early fruit development physiological measurements are also needed to validate the proposed source–sink regulatory mechanism.

## 4. Materials and Methods

### 4.1. Experimental Site

**Figure 7:**
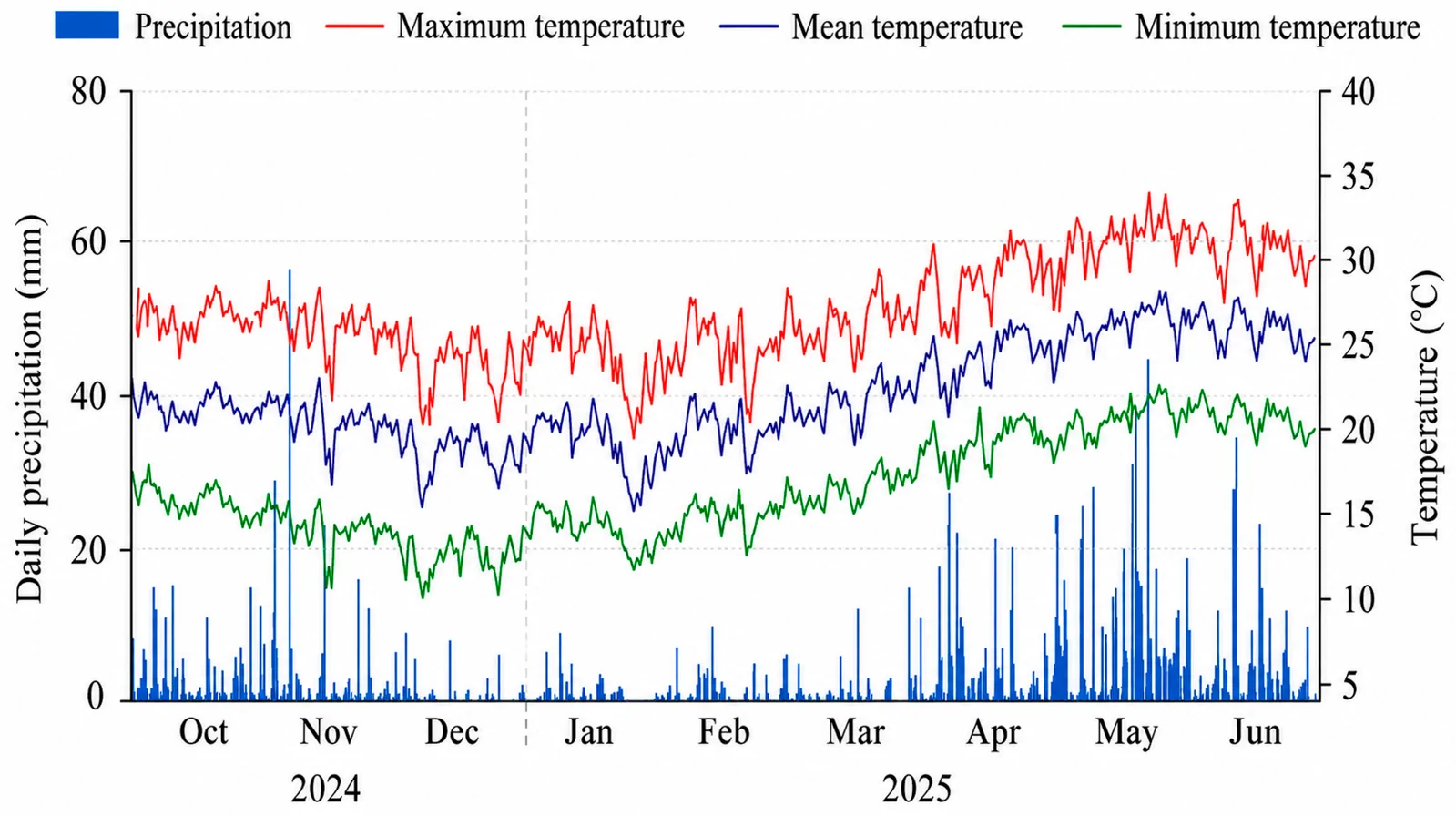
Climatic conditions during the field experiment, showing daily precipitation and daily maximum, mean, and minimum air temperatures from October 2024 to June 2025.

The experiment was conducted in a commercial macadamia orchard located in Nannuoshan Village, Menghai County, Xishuangbanna Prefecture, Yunnan Province, China. The orchard consisted of 9-year-old trees planted at a spacing of 4.0 m × 6.0 m. The orchard covered approximately 167 ha and was situated at an elevation of approximately 1250 m above sea level on slopes ranging from 6° to 15°. The soil was classified as red soil. The physicochemical properties of the 0–20 cm soil layer were as follows: organic matter, 18.20 g kg^−1^; total nitrogen, 1.39 g kg^−1^; available phosphorus, 17.04 mg kg^−1^; available potassium, 133.32 mg kg^−1^; and pH 5.40. Daily precipitation and air temperature during the experimental period are shown in Figure 7.

To characterize the meteorological conditions around each deflowering and tagging date, temperature and precipitation were summarized over four 15-day windows, each extending from seven days before to seven days after the operation date (Table 3). Mean air temperature was calculated as the average of the daily mean temperatures within each window. Minimum and maximum temperatures were obtained from the hourly observations, and cumulative precipitation was calculated by summing hourly precipitation amounts. These standardized windows were used to describe conditions around treatment application and were not intended to represent the full duration of each flowering flush.

### 4.2. Plant Materials

Seed-propagated macadamia trees were used as the experimental material. The experimental trees were obtained from a commercially established orchard with uniform planting management. The trees were non-grafted and grown on their own roots; therefore, no rootstock was used. At the time of the experiment, they were nine years old and approximately 6 m tall, with canopy diameters of approximately 7 m in the north–south direction and 6 m in the east–west direction. Trees with similar vigor, canopy characteristics, and previous-year productivity were selected before treatment application. Trees showing visible symptoms of pests or diseases were excluded. During the experiment, all trees were managed according to local commercial production practices, including irrigation, fertilization, pest control, and pruning. Except for the flower-removal treatments, all management practices were consistent among treatments.

### 4.3. Experimental Design

Field observations indicated that cultivar ‘O.C’ exhibited multiple flowering flushes during the study period, with major flowering events occurring on 25 October 2024, 7 January 2025, 13 February 2025, and 24 March 2025. These four flowering flushes were selected on the basis of the flowering pattern observed in the experimental orchard. October represented off-season flowering before the main flowering season, whereas January, February, and March represented successive flushes. Comparing the removal of these distinct cohorts allowed us to examine how flower-removal timing affected the remaining reproductive organs and whole-tree yield. Five treatments were established according to the flowering period removed: A (CK), no flower removal; B, removal of October off-season flowers; C, removal of January flowers; D, removal of February flowers; and E, removal of March flowers.

A randomized complete block design was adopted with five blocks. Twenty-five trees with similar growth vigor and canopy characteristics were selected, and one tree from each treatment was randomly assigned within each block, resulting in five biological replicates per treatment. Flower removal was conducted manually at the beginning of full bloom for the target flowering period. All inflorescences belonging to the target flowering flush were removed from the whole tree, whereas inflorescences from other flowering periods were retained.

For each flowering flush, 100 inflorescences per tree were selected and tagged on the outer-middle canopy before the corresponding flower-removal operation. Tagging was conducted separately for the October, January, February, and March flushes. In each removal treatment, all inflorescences of the designated flush, including those already tagged, were removed from the whole tree. Subsequent observations followed the same tagged inflorescences within the retained flowering flushes. At each assessment, the number of attached nutlets on each monitored inflorescence was recorded, and an inflorescence was classified as fruit-bearing if at least one fruit remained attached.

### 4.4. Measurements and Analytical Methods

#### 4.4.1. Inflorescence Retention and Nutlet Abscission

Tagged inflorescences from retained flowering cohorts were monitored throughout fruit development. At each assessment, an inflorescence was classified as fruit-bearing if at least one developing fruit remained attached. The initial fruit-bearing inflorescence percentage was assessed at 10 days after flowering, and the final fruit-bearing inflorescence percentage was assessed before harvest. These variables describe retention at the inflorescence level and do not represent the percentage of individual flowers that set fruit.

The initial fruit-bearing inflorescence was calculated according to Equation (1):

(1) $$\mathrm{Initial}\;\text{fruit-bearing}\;\mathrm{inflorescences}\;\left(\%\right)=\frac{I_{initial}}{I_{tagged}}\times 100$$

The final fruit-bearing inflorescence was calculated according to Equation (2):

(2) $$\mathrm{Final}\;\text{fruit-bearing}\;\mathrm{inflorescences}\;\left(\%\right)=\frac{I_{final}}{I_{tagged}}\times 100$$

Here, *I_tagged_* is the number of tagged inflorescences monitored in a retained cohort, and *I_initial_* and *I_final_* are the numbers bearing at least one fruit at the initial and final assessments, respectively. Inflorescences intentionally removed by treatment were excluded from these calculations.

The relative loss of fruit-bearing inflorescences between the initial and final assessments was calculated as:

(3) $$\text{Fruit-bearing}\;\mathrm{inflorescence}\;\mathrm{loss}\;\left(\%\right)=\frac{I_{initial}-I_{final}}{I_{initial}}\times 100$$

This measure describes the loss of fruit-bearing status among inflorescences and does not quantify the number of individual nutlets abscised. To assess nutlet abscission separately, the actual number of attached nutlets on the same tagged inflorescences was recorded at successive assessments. Cumulative nutlet abscission was calculated as:

(4) $$\mathrm{Cumulative}\;\mathrm{nutlet}\;\mathrm{abscission}\;\left(\%\right)=\frac{N_{0}-N_{t}}{N_{0}}\times 100$$

Here, *N*_0_ is the number of nutlets at the initial counting assessment, and *N_t_* is the number remaining on the same tagged inflorescences at assessment *t*.

#### 4.4.2. Fruit Growth Dynamics and Logistic Model Fitting

Fruit samples were collected at 20, 40, 60, 80, 100, 120, 140, 160, and 180 days after flowering (DAF). For each treatment, five trees were used as biological replicates. At each sampling time, 10 healthy and uniformly developed fruits were randomly collected from each tree, resulting in 50 fruits per treatment. The mean fruit fresh weight of each tree was calculated and used as the biological replicate for statistical analysis and Logistic model fitting.

Fresh weight was determined using an analytical balance (Quintix 224-1S, Sartorius, Göttingen, Germany). Fruit growth curves were fitted based on the mean fruit fresh weight of each tree using a Logistic growth model:

(5) $$W(t)=\frac{W_{\max}}{1+\exp(-k(t-t_{0}))}$$

where *W*(*t*) is the fruit fresh weight at time t, *W_max_* is the theoretical maximum fruit weight, k is the growth rate constant, and *t*_0_ is the inflection point of fruit growth.

The parameters *W_max_*, *k*, and *t*_0_ were estimated separately for each tree. Treatment-level parameter estimates were summarized as means ± standard errors (SE) across five trees per treatment. SE was used to describe the uncertainty of the mean parameter estimates. The coefficient of determination (R^2^) was used to describe model fit.

#### 4.4.3. Fruit Morphology, Kernel Characteristics, and Nutritional Quality

At maturity, 10 healthy fruits of uniform size were collected from each of five trees per treatment, giving 50 fruits per treatment. Samples were collected from comparable fruiting shoots and canopy positions across treatments to minimize microenvironmental variation. Fruits were sampled from the outer-middle canopy across the four cardinal directions (east, west, south, and north). Samples originated from the January, February, and March flowering flushes retained under each treatment. Each tree was treated as a biological replicate.

Fruit samples were collected from comparable fruiting shoots and canopy positions among treatments to minimize variation caused by microenvironmental differences.

Fruit transverse diameter and longitudinal diameter were measured using a digital caliper (500-196-30, Mitutoyo, Kawasaki, Japan). Dry nut weight and kernel dry weight were determined after shell removal.

Kernel recovery was calculated according to Equation (6):

(6) $$\mathrm{Kernel}\;\mathrm{recovery}\;(\%)=\left(\frac{\mathrm{Kernel}\;\mathrm{dry}\;\mathrm{weight}}{\mathrm{Dry}\;\mathrm{nut}\;\mathrm{weight}}\right)\times 100$$

Kernel crude fat, protein, and total sugar contents were determined using a near-infrared spectroscopy analyzer (NIRS™ DS2500, FOSS, Hillerød, Denmark) equipped with validated calibration models supplied and maintained according to the manufacturer’s recommendations.

At harvest, the total fresh fruit yield of each experimental tree was recorded with the green husk intact and expressed as kg tree^−1^. Yield included harvested fruits from all retained flowering cohorts. Each tree was treated as a biological replicate, and yield was summarized as mean ± SE across five trees per treatment. Harvest totals were not recorded separately for individual flowering cohorts.

#### 4.4.4. Determination of Source-Related Traits

Leaf and branch samples were collected during the fruit enlargement stage, at 120 days after flowering (DAF), separately for each retained January, February, and March flowering flush. DAF was referenced to the peak flowering date of the corresponding flush. Mature functional leaves were sampled from the middle-upper portion of tagged fruit-bearing shoots in the outer-middle canopy, and corresponding branch segments were collected simultaneously from the same shoots. Samples were collected from comparable canopy positions and orientations across treatments. For each tree, 20 leaves and five current-year branch segments, each approximately 15 cm long, were collected from the outer-middle canopy across the four cardinal directions. Samples from the same tree were combined into a single composite sample per tissue type. Five trees per treatment were used as biological replicates.

Mature functional leaves were sampled from the middle-upper portion of tagged fruit-bearing shoots in the outer-middle canopy, and the corresponding fruit-bearing branch segments were collected simultaneously from the same shoots. Samples were collected from comparable canopy positions and orientations among treatments to minimize environmental variation.

Leaf chlorophyll status was estimated using a portable chlorophyll meter (SPAD-502 Plus, Konica Minolta, Tokyo, Japan). Five readings were taken per leaf and averaged.

Soluble sugar concentrations in leaves and branches were determined using the anthrone colorimetric method; Starch content was measured after acid hydrolysis followed by colorimetric analysis [45]. Absorbance was measured using a UV–Vis–NIR spectrophotometer (Cary 5000, Agilent Technologies, Santa Clara, CA, USA). Total nitrogen content was determined by the Kjeldahl method following H_2_SO_4_–H_2_O_2_ digestion [46], using a DKL-series digestion unit and a UDK-series Kjeldahl distillation unit (VELP Scientifica, Usmate, Italy).

To evaluate the balance between carbohydrate accumulation and nitrogen status, the carbon-to-nitrogen ratio (C/N ratio) was calculated according to Equation (7):

(7) $$C/N\;\mathrm{ratio}=\frac{\mathrm{Soluble}\;\mathrm{sugar}+\mathrm{Starch}}{\mathrm{Total}\;\mathrm{nitrogen}}$$

#### 4.4.5. Descriptive Indices of Carbohydrate and Nitrogen Status

Two exploratory indices, the branch–leaf carbohydrate index (BCI) and the carbon–nitrogen index (CNI), were calculated to summarize the measured carbohydrate and nitrogen status of leaves and branches. BCI combines soluble sugar and starch concentrations in both tissues to provide a relative description of carbohydrate status within the study dataset. CNI expresses the average of these four carbohydrate measurements relative to the mean total nitrogen concentration of the two tissues. These indices were used alongside the individual measurements to describe treatment-associated variation.

BCI was calculated as follows:

(8) $$BCI=\frac{100}{4}\left(\frac{LSS}{LSS_{max}}+\frac{LST}{LST_{max}}+\frac{BSS}{BSS_{max}}+\frac{BST}{BST_{max}}\right)$$

where LSS, LST, BSS, and BST represent leaf soluble sugar, leaf starch, branch soluble sugar, and branch starch concentrations, respectively. The subscript “max” denotes the maximum value of each variable across treatment means in the study dataset. Normalization places the four variables on a comparable numerical scale. The normalized components were assigned equal weights, and their mean was multiplied by 100. BCI is therefore a dimensionless index expressed on a 0–100 scale, rather than a measured carbohydrate percentage. Equal weighting was adopted as a descriptive convention and does not imply equal physiological contributions of the four components.

CNI was calculated as follows:

(9) $$CNI=\frac{\left(LSS+LST+BSS+BST\right)/4}{TN_{mean}}\;{TN}_{mean}=\frac{TN_{leaf}+TN_{branch}}{2}$$

where *TN_l_*_eaf_ and *TN_branch_* represent leaf and branch total nitrogen concentrations, respectively. All carbohydrate and nitrogen concentrations were expressed in the same units and on the same mass basis before calculation. CNI is dimensionless and represents a composite carbohydrate-to-nitrogen index; it is not the ratio of total carbon to total nitrogen.

Higher BCI values indicate higher average normalized carbohydrate concentrations, whereas higher CNI values indicate a higher composite carbohydrate concentration relative to mean tissue nitrogen concentration. Neither index accounts for the relative biomass of leaves and branches, and neither estimates whole-tree carbohydrate reserves. Their interpretation depends on the selected variables, normalization procedure, and sampling stage. They do not directly measure assimilate transport, sink demand, or whole-tree source–sink balance and have not been independently validated as predictors of fruit quality or yield.

### 4.5. Statistical Analysis

All data were compiled using Microsoft Excel 2021 and analyzed using SPSS 26.0 (IBM Corp., Armonk, NY, USA) and R version 4.3.3 (R Foundation for Statistical Computing, Vienna, Austria). Each tree was considered as the biological replicate. Treatment effects were evaluated using one-way analysis of variance (ANOVA), followed by Tukey’s honestly significant difference (HSD) test at *p* < 0.05.

Fruit retention dynamics were analyzed using linear mixed-effects models, with treatment and developmental stage as fixed effects and tree as a random effect. Fruit growth curves were fitted using nonlinear regression based on the Logistic model. The parameters *W_max_*, *k*, and *t*_0_ were estimated for each tree and summarized as treatment means ± SE (*n* = 5 trees per treatment).

Pearson correlation analysis was performed to evaluate relationships between source-related traits and fruit characteristics. Partial least squares (PLS) regression was conducted as an exploratory multivariate analysis, with source-related traits as explanatory variables (X-matrix) and fruit characteristics as response variables (Y-matrix). Variable importance in projection (VIP) scores were used to summarize the relative contribution of explanatory variables within the fitted model. Variables with VIP values > 1.0 were considered relatively important contributors. VIP scores were interpreted as exploratory measures of variable importance rather than evidence of predictive performance.

Principal component analysis (PCA) was performed using standardized variables, including fruit retention traits, fruit quality traits, source-related traits, and the composite parameters BCI and CNI, to visualize multivariate differences among treatments.

All figures were generated using Origin 2023 (OriginLab Corp., Northampton, MA, USA) and R software.

## 5. Conclusions

Staged flower removal altered fruit-bearing raceme persistence, nutlet abscission, individual fruit growth, kernel quality, and source-related carbohydrate status in seed-propagated macadamia trees. Removal of October off-season inflorescences increased final retention of fruit-bearing tagged inflorescences from 12.9% to 18.3%, individual fruit fresh weight at 180 days after flowering from 20.1 to 25.5 g, and kernel dry weight from 1.48 to 1.73 g. However, mean whole-tree fresh nut-in-husk yield was 35.98 kg tree^−1^, compared with 54.75 kg tree^−1^ in the control. Improvements in the retained inflorescences and sampled fruits therefore did not translate into higher mean whole-tree yield.

The accompanying changes in leaf and branch carbohydrate status were associated with fruit responses but did not establish a causal mechanism. BCI and CNI remain exploratory indices requiring independent validation. Given the single-site, single-season scope, the lower mean yield under October removal, and the absence of an economic assessment, complete removal of October inflorescences cannot currently be recommended for commercial production. Further studies should evaluate partial flower removal, marketable kernel yield, labor costs, and subsequent-season production.

## Figures and Tables

**Table 1:** Reproductive characteristics of different flowering flushes in macadamia cultivar ‘O.C’.

Flowering Flush	Fruit Set per Inflorescence (%)	Average Retained Nuts per Inflorescence	Inflorescences Bearing One Nut (%)	Inflorescences Bearing 2–4 Nuts (%)	Inflorescences Bearing ≥ 5 Nuts (%)
October	0.63 ± 0.06 a	4.10 ± 0.17 a	22.6 ± 2.8 c	59.1 ± 4.3 a	18.3 ± 3.1 a
January	0.33 ± 0.02 b	1.23 ± 0.05 c	80.2 ± 5.6 a	18.6 ± 2.6 b	1.2 ± 0.1 d
February	0.30 ± 0.03 b	2.12 ± 0.23 b	36.6 ± 4.1 b	57.0 ± 5.2 a	6.4 ± 2.1 c
March	0.19 ± 0.07 c	2.43 ± 0.34 b	41.7 ± 4.7 b	45.5 ± 6.3 a	12.8 ± 3.4 b

Note: Values are presented as mean ± SE. Different lowercase letters within the same column indicate significant differences among flowering flushes according to Tukey’s test (*p* < 0.05).

**Table 2:** Parameters of logistic growth models describing fruit fresh weight accumulation under different flower-removal treatments in macadamia cultivar ‘O.C’.

Treatment	*W_max_* (g)	*k* (d^−1^)	*t*_0_ (DAF)	R^2^
A(CK)	20.5 ± 0.6 c	0.043 ± 0.002 c	88.5 ± 2.1 a	0.982
B	25.6 ± 0.7 a	0.056 ± 0.003 a	78.5 ± 1.8 c	0.994
C	21.4 ± 0.5 bc	0.047 ±0.002 bc	84.0 ±2.0 b	0.987
D	22.2 ±0.6 b	0.051 ±0.002 b	81.5 ±1.9 bc	0.989
E	21.0 ±0.5 c	0.040 ±0.002 c	90.8 ±2.4 a	0.981

Note: *W_max_*, *k*, and *t*_0_ are presented as means ± SE of parameter estimates obtained from five individual-tree growth curves per treatment (*n* = 5 trees). Different lowercase letters indicate significant differences among treatments according to Tukey’s HSD test (*p* < 0.05). R^2^ denotes the coefficient of determination.

**Table 3:** Meteorological summaries for the four 15-day windows centred on the deflowering and tagging dates.

Date Window	Mean Air Temperature (°C)	Minimum Air Temperature (°C)	Maximum Air Temperature (°C)	Cumulative Precipitation (mm)
18 October–1 November 2024	22.20	16.51	29.70	23.16
31 December 2024–14 January 2025	13.85	6.89	23.99	2.87
6–20 February 2025	18.36	9.31	29.46	1.15
17–31 March 2025	19.49	10.43	33.84	9.13

Note: Each window includes the operation date and the seven days preceding and following it. Mean air temperature is the average of the 15 daily mean temperatures. Minimum and maximum temperatures are the lowest and highest hourly air temperatures recorded within each window, respectively. Cumulative precipitation is the sum of hourly precipitation amounts within the window. The operation dates were 25 October 2024, 7 January 2025, 13 February 2025, and 24 March 2025. These windows describe meteorological conditions around treatment application and do not represent the full duration of each flowering flush.

## Data Availability

The data that support the findings of this study are available from the corresponding author upon reasonable request.
